# Ethanol induces cytostasis of cortical basal progenitors

**DOI:** 10.1186/s12929-016-0225-8

**Published:** 2016-01-19

**Authors:** Amanjot Kaur Riar, Madhusudhanan Narasimhan, Mary Latha Rathinam, George I. Henderson, Lenin Mahimainathan

**Affiliations:** Department of Pharmacology and Neuroscience, Texas Tech University Health Sciences Center, 3601 4th Street, Lubbock, TX 79430 USA; South Plains Alcohol and Addiction Research Center, Texas Tech University Health Sciences Center, 3601 4th Street, Lubbock, TX 79430 USA

**Keywords:** Fetal alcohol syndrome, basal progenitors, cyclin D1

## Abstract

**Background:**

Developing brain is a major target for alcohol’s actions and neurological/functional abnormalities include microencephaly, reduced frontal cortex, mental retardation and attention-deficits. Previous studies have shown that ethanol altered the lateral ventricular neuroepithelial cell proliferation. However, the effect of ethanol on subventricular basal progenitors which generate majority of the cortical layers is not known.

**Methods:**

We utilized spontaneously immortalized rat brain neuroblasts obtained from cultures of 18-day-old fetal rat cerebral cortices using in vitro ethanol exposures and an in utero binge model. In the in vitro acute model, cells were exposed to 86 mM ethanol for 8, 12 and 24 h. The second in vitro model comprised of chronic intermittent ethanol (CIE) exposure which consisted of 14 h of ethanol treatment followed by 10 h of withdrawal with three repetitions.

**Results:**

E18 neuroblasts expressing Tbr2 representing immature basal progenitors displayed significant reduction of proliferation in response to ethanol in both the models. The decreased proliferation was accompanied by absence of apoptosis or autophagy as illustrated by FACS analysis and expression of apoptotic and autophagic markers. The BrdU incorporation assay indicated that ethanol enhanced the accumulation of cells at G1 with reduced cell number in S phase. In addition, the ethanol-inhibited basal neuroblasts proliferation was connected to decrease in cyclin D1 and Rb phosphorylation indicating cell cycle arrest. Further, in utero ethanol exposure in pregnant rats during E15-E18 significantly decreased Tbr2 and cyclin D1 positive cell number in cerebral cortex of embryos as assessed by cell sorting analysis by flow cytometry.

**Conclusions:**

Altogether, the current findings demonstrate that ethanol impacts the expansion of basal progenitors by inducing cytostasis that might explain the anomalies of cortico-cerebral development associated with fetal alcohol syndrome.

**Electronic supplementary material:**

The online version of this article (doi:10.1186/s12929-016-0225-8) contains supplementary material, which is available to authorized users.

## Background

Maternal alcohol use during pregnancy induces grave abnormalities and deficiencies in developing fetus termed as fetal alcohol spectrum disorder (FASD) [1–3], one of the leading and preventable global health problems. Permanent central nervous system dysfunction is the most severe characteristic of fetal alcohol syndrome. Even a small amount of alcohol consumed during gestation inflicts adverse to profound brain damage to developing fetus that is dependent on the stage/phase of pregnancy [4]. Intrauterine ethanol exposure damages developing brain cells (such as neural crest, neural progenitors, radial glia and newly born neurons) causing structural brain malformations leading to several functional, behavioral and cognitive impairments including but not limited to poor memory, hyperactivity, attention deficits, poor judgment and reasoning, learning problems and impulsivity [5–7]. Alcohol exerts its teratogenic effects on various parts of the developing brain including cerebral cortex, cerebellum, basal ganglia, corpus callosum and hippocampus. The developing cerebral cortex is particularly sensitive to ethanol toxicity [8, 9]. Studies have shown that prenatal ethanol exposure during the sensitive period of cortex development decreases the genesis of cortical neurons as well as the demise of newly born neurons ensuing disproportionate reduction in the size of the cortex [9, 10].

The ontogenetic development of 6-layered cerebral cortex is a multifaceted, dynamic and tightly controlled process arises from dorsal part of telencephalon. The six layers are generated from the projection neurons arising from ventricular zone (VZ) and subventricular zone (SVZ) in an inside-outside fashion as evidenced from rodent studies [11]. Although cortical lamination and fundamental neuronal cell types are largely conserved, they differ in laminar thickness, neuron density, its subtype, size, shape, connectivity and function. Multipotent neuroepithelial cells (NECs) often known as “neural stem cell (NSC)” of lateral ventricles generates the cerebral cortex virtually in all vertebrate species [12]. As corticogenesis proceeds, NECs give rise to preplate neurons, radial glial progenitors (RGPs), short neural precursors (SNPs) and intermediate neuronal progenitor (INPs) commonly known as basal progenitors (BPs) [12–17]. Scientific literature often refers precursor/progenitor as “neuroblasts”. NECs and RGPs both express transcription factor Pax6 and intermediate filament protein nestin although RGPs also express astroglial markers such as vimentin and brain lipid binding protein (BLBP) [18]. RGPs, more fate restricted cell, at the apical surface undergo symmetric as well as asymmetric division to produce another radial glial cell or neuron or alternatively to basal progenitors at defined distance in SVZ. Basal progenitors lacking polarity and interkinetic nuclear migration exclusively give rise to neurons, implying its crucial role in the amplification of projection neurons from neuroepithelial and radial glial cell. One of the characteristic of BPs is that their division occurs in the basal surface and NECs and RGPs divide at the apical surface [15–19]. During the onset of corticogenesis, BPs delaminates from the VZ and propagates into the SVZ. The formation of SVZ begins at embryonic day 13.5 in mouse and enlarges substantially throughout late corticogenesis. Unlike apical progenitors in VZ, BPs lack Pax6 but exclusively express transcription factor T-box brain protein 2 (Tbr2)/ EOMES [20] and undergoes only symmetric division giving rise to two neurons. Although Tbr2 positive-BPs are thought to predominantly generate upper cortical layer neurons [21, 22], evidences suggest that they also generate deep cortical layer neurons [13, 16, 23].

Notably, much of the research in FASD has focused on apical progenitors: neural stem cell and radial glia cells and several mechanisms including defective proliferation, disrupted cell cycle events, decreased neurogenesis, differentiation and increased apoptosis have been uncovered explaining the teratogenic effects of ethanol on this population of cells [20–28]. Although the existing few studies on BPs demonstrate an emerging trend about their role in the development of massive cortical surface expansion, gyrification and laminar patterning [24, 25], research pertaining to effects of alcohol and the mechanisms underlying altered corticogenesis in BPs are still scant and unclear. Thus the objective of the current study is to examine the effect of ethanol on Tbr2- (+)ve-BP cell proliferation and associated cell fate mechanism leading to neurodevelopmental anomalies.

## Methods

### Materials, antibodies and other reagents

Ham’s F-12 medium, L-glutamine, tubulin, NF-200, 5-bromo-2’deoxyuridine (BrdU), dibutyryl cyclic adenosine monophosphate sodium salt (cAMP) were purchased from Sigma-Aldrich (St. Louis, MO). Fetal bovine serum (FBS) (Atlanta Biologicals, Lawrenceville, GA), antibiotic-antimycotic, trypsin-EDTA, (Gibco, Grand Island, NY), propidium iodide (PI), mouse IgG AB:FITC, anti-bromodeoxyuridine antibody were from (Millipore, Billerica, MA), antibodies for beclin-1, phospho-retinoblastoma (ser 795) were from (Cell Signaling Technology, Beverly, MA), glyceraldehyde 3-phosphate dehydrogenase (GAPDH), caspase-3, cyclin D1, T-box-brain2 (Tbr2)/Eomesodermin (EOMES), goat-anti-mouse IgG-HRP, goat anti-rabbit IgG-HRP were from (Santa Cruz Biotechnologies, Santa Cruz, CA) and NSE was from Polysciences, Inc. Warrington, PA. All other reagents were obtained from different manufacturers and were of highest grade.

### Cell culture

We utilized spontaneously immortalized rat brain neuroblasts obtained from cerebral cortices of embryonic day-18 (E18)- fetus. These cells were generously provided by Dr. Alberto Muñoz (Instituto de Investigaciones Biomédicas, CSIC, Madrid, Spain). Cells were cultured in Ham’s F-12 media enriched with 10 % FBS, L-glutamine (2 mM), streptomycin (100 μg/ml), penicillin (100 units/ml) and plasmocin (5 μg/ml). Cells were grown in the humidified incubator maintained at 37^0^ C with 95 % air and 5 % CO_2._ Early passage of neuroblasts was used throughout the study.

### Ethanol (ETOH) treatment

Experiments involving acute model of ETOH in vitro in the current literature spans from minutes to hours uses a concentration of 1–500 mM [26] with a dose above 100 mM has been used to study the cytotoxic effect and related mechanisms of ETOH toxicity [27]. Ethanol concentration ranging from 10–100 mM for 24 h has been largely regarded as physiological in *in vitro* studies, though 25 mM concentration being close to 0.08 % blood alcohol level achieved by human consuming 4-5 drinks. Hence in the current study we used physiologically relevant ETOH concentrations of 2.5 mg/ml and 4 mg/ml corresponding to ~54 mM and ~ 86 mM respectively. ETOH treatments were performed in a separate incubator previously saturated with 100 % (200 proof) ethanol in order to maintain the ETOH concentration at the level added to the media [28]. Further, ETOH concentration was regularly monitored using Analox AM1 alcohol analyzer (Analox Instruments, MA, USA) [29]. Control cells were maintained in the ethanol-free incubator.

### Acute and chronic intermittent ethanol exposure paradigm

Two different models of ethanol exposures, acute exposure and chronic intermittent ethanol exposure (CIE) were used. In the acute paradigm, cells were treated with or without 4 mg/ml (86 mM) ETOH for 8, 12 and 24 h; whereas in the CIE paradigm cells were exposed to either 2.5 mg/ml or 4 mg/ml ETOH for three cycles, each cycle of 14 h of ETOH treatment followed by 10 h of withdrawal. During the withdrawal phase media containing ETOH was removed and replaced with fresh media and kept in the ETOH-free incubator. Controls were also subjected to similar media changes. Cells were harvested in the last cycle after 14 h of ETOH treatment [30].

### In vivo model

Pregnant Sprague Dawley rats at gestation day 15 were administered with ETOH (3.5 g/kg body weight, 25 % v/v) at 12 h intervals for 3 days. This acute ethanol exposure regimen in an animal model mimics binge drinking in humans [31]. Pair-fed control rats were weight matched to the ETOH-fed dams and was intubated with iso-caloric dextrose. Both iso-caloric dextrose intubated control and ETOH-fed dams had full access to water, whereas pair-fed controls received the weight of chow consumed by the corresponding ethanol dam during the previous 24 h period. At the end of the treatment, pregnant rats were sacrificed by decapitation and blood alcohol levels were determined using Analox AM1 analyzer. Fetal brains were isolated, cerebral cortices were dissected out and the tissues were isolated into single cells by mechanical disruption and processed for FACS analysis. All animals were maintained in accordance with Institutional Animal Care and Use Committee-approved procedures bearing the protocol number, 10029.

### Assessment of proliferation index by cell counting

Confluent cells were treated in the presence or absence of 4 mg/ml ETOH for 8, 12 and 24 h or subjected to CIE regimen as described above. After treatment, cells were briefly washed in 1 X PBS and detached by adding 0.5 ml of 0.25 % trypsin for 1–2 min which was immediately followed by a termination reaction with 0.5 ml of FBS containing media. 0.5 ml of suspension from each well was quantified for viable cells and percentage viability using Vi-CELL analyzer. Experiments were also replicated in different passages.

### Assessment of proliferation index by 5-bromo-2’deoxyuridine (BrdU) incorporation

Cells at a confluency of 75–80 % were treated with 4 mg/ml ETOH for 24 h. 4 h prior to harvest, cells were pulse labeled with 30 μM BrdU in dark. After labeling and at the end of the experiment, cells were detached by trypsinization, washed and fixed in 0.7 ml ice-cold 100 % ethanol. Cells were then centrifuged and PBS containing 0.5 mg/ml RNase A (Sigma) was added to the pellet and incubated at 37^0^ C for 30 min. Permeabilization of cells was done by treatment with 1 ml of 2 N HCl-Triton solution on ice for 10 min followed by incubation at 97^0^ C for 15 min. Cells were incubated for 1 h at room temperature with anti-BrdU antibody (1:100 dilution) followed by addition of fluorescein isothiocyanate (FITC) –conjugated antibody for 30 min. Finally, cells were centrifuged at 1150xg for 2 min and resuspended in 40 μg propidium iodide (PI) in dark. Samples were covered in aluminium foil until flow cytometric analysis was performed. Samples with no BrdU and propidium iodide were included in the experiments as negative control to establish the validity of staining. Analysis was performed by two gated flow cytometer (BD LSR II Flow Cytometer, Biosciences) using FACSDiva software. Only singlet cells were considered for analysis.

### Cytotoxicity assay

To assess the effect of ETOH on cell cytotoxicity, we used ApoTox-Glo Triplex assay (Promega, Madison, WI). Briefly 1 X 10^4^ cells/well were seeded in a 96 well plate and treated with or without 4 mg/ml ETOH for 24 h. Subsequently 10 μl of viability/cytotoxicity reagent (provided with the kit) was added to the wells and mixed by orbital shaking at 500 rpm for 30 s. Plates were incubated at 37^0^ C for 45 min and fluorescence was measured at 485_Ex_/520_Em_. Negative controls including wells with no cells and with no reagent was used in the experiment.

### Protein extraction, determination and Western blotting

Briefly, following the experiments, cells were washed in 1X PBS and lysed in radio-immunoprecipitation assay (RIPA) buffer supplemented with 1 X protease inhibitor cocktail (Sigma), sonicated (Sonics, vibra-cell ultrasonic processor) for 5 s at an amplitude of 25 % and centrifuged at 21,900xg for 20 min at 4 °C. Clarified supernatants were then subjected for protein measurement by Biorad Spectrophotometer at 450 nM using Bio-Rad reagent protein assay dye reagent (Bio-Rad Laboratories). 30 μg protein was mixed with SDS lysis buffer and denatured at 95^0^ C for 6 min. The samples were then separated by sodium dodecyl sulfate polyacrylamide gel electrophoresis (SDS-PAGE) and transferred to polyvinylidene (PVDF) membrane (Bio- Rad, CA). Non-specific binding was blocked by 5 % nonfat dry milk powder in PBST. Membranes were then incubated with primary antibodies against NSE, NF -200, TBR2/EOMES, caspase 3, beclin, phospho-Rb (ser 795), Cyclin D1, GAPDH and tubulin in either 1:1000 or 1:500 dilution for 3 h or overnight. After 3 washes with PBST, membranes were incubated with suitable anti-rabbit or anti- mouse IgG secondary antibody conjugated with horseradish peroxidase in a dilution of 1:10000 for 1 h. Blots were washed in PBST and were developed with ECL chemiluminescence Western blot kit (Thermo scientific, IL, USA) and the immunoreactive signals were captured onto a HyBlot CL Autoradiography Film (Denville Scientific, Metuchen, NJ). The detected signals were scanned using Adobe Photoshop CS2 and quantitated using Scion Image software (Scion Corporation). The relative intensity of bands was normalized to the housekeeping controls, GAPDH or Tubulin.

### Cell cycle analysis

Cells were treated with or without 4 mg/ml ETOH for 24 h in a 6 well plate. After treatment, media was removed from the plates, washed in 1X PBS and harvested by gentle trypsinization followed by centrifugation at 160xg at 4^0^ C for 5 min. Supernatant was decanted and the pellets were resuspended in PBS. Cells were then counted and 1 × 10^6^ cells were transfered to FACS tube and washed by centrifugation in 1 ml of PBS. Pelleted cells were resuspended in 0.3 ml of PBS and were fixed by dropwise addition of 0.7 ml of 100 % cold ethanol followed by 1 h incubation on ice. Cells were then washed and resuspended in 0.25 ml of PBS followed by addition of 5 μl of 10 mg/ml RNase A (Sigma). After incubation at 37^0^ C for 1 h, cells were resuspended with 40 μg/ml of propidium iodide and analyzed by flow cytometry (BD LSR II Flow Cytometer) at 488 nm.

### Analysis of Tbr2 and Cyclin D1 expressing cells using multi-color and multi-parametric flow cytometry

Dissociated cerebral cortical cells were washed with PBS, fixed using ice-cold 100 % methanol and stored in -80^0^ C for 48 h. For staining, 1 × 10^6^ cells per group (n ≥ 3) were rinsed with FACS buffer (0.2 % BSA in PBS) and incubated with anti-Tbr2-PE (1 μg/ul) and anti-Cyclin D1- FITC (1 μg/ul) conjugated antibody for 45 min in dark. The respective isotype control antibodies were used at the same concentrations. Cells were rinsed twice and resuspended in FACS buffer prior to analysis with flow cytometry (BD FACSAriaII). Data was acquired and analyzed using FACSDiva and FlowJo softwares as described previously [32, 33]. Baseline PMT voltages were optimized using unstained cells and isotype controls. All experiments were performed by employing compensation for spillover of FITC over PE. Two-parameter cytogram of side scatter and forward scatter profiles were used to eliminate debris and doublet cells. Tbr2-positive cells were identified by gating on the single-parameter PE histogram. Tbr2-positive cells were further analyzed for the expression of cyclin D1 by gating on single-parameter FITC histogram. The percent positive cells in each condition were determined by gating of PE and FITC histogram and their respective isotype controls.

### Statistical analysis

All results are shown as mean ± SEM. For comparing more than two groups, one way analysis of variance (ANOVA) followed by Student–Newman–Keul’s post hoc analysis was used to determine statistical significance. Student’s *t*-test was used for experiments involving only two groups. *p* < 0.05 was considered as statistically significant. All statistical analysis was conducted using GraphPad Instat software.

## Results

### E18 neuroblasts represent immature basal progenitor which on differentiation express neuronal markers

**Fig. 1:**
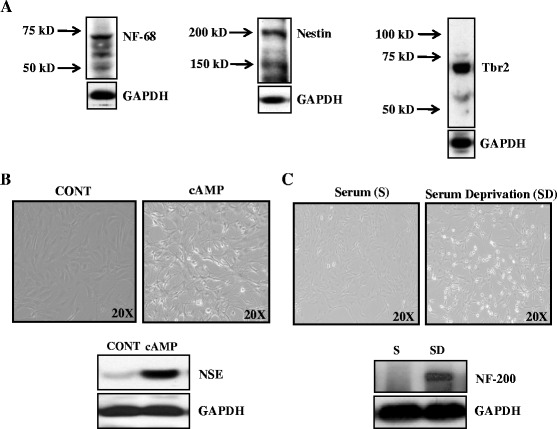
Characterization of cortical neuroblasts. We characterized the cells with various immature and mature neuronal markers. Western blot analysis demonstrated that the cells expressed Tbr2, nestin and NF-68 and lacked NSE and NF-200 indicating primitive nature of cells [Fig. 1**a**, (**b**, lower panel, first lane) & (**c**, lower panel, first lane)]. Application of cAMP for 24 h stimulated neuronal differentiation which is apparent by neurite outgrowth. (Fig. 1**b**, right). Further differentiation induction with cAMP showed the presence of neuron marker NSE compared to control (CONT) (Fig. 1**b**, lower panel). The classical neural differentiation potential of these nestin-positive cells was further determined by maintaining the cells in serum free media. It is known that serum deprivation promotes neural differentiation. Most of the cells after 24 h of serum deprivation induced differentiation which was evident by neuron like morphology with spherical cell body and long processes (Fig. 1**c**, right). Neuronal marker, NF-200 which is generally expressed after differentiation was confirmed by NF-200 immunoblotting (Fig. 1**c**, lower panel)

To ensure that the cells used were of neuroblastic phenotype and exhibit respective characteristics, early passage of cells used throughout the study along with performing an initial characterization for various immature and mature neuronal markers. Cells expressed nestin and NF-68 and lacked NSE and NF-200 indicating primitive nature of cells [Fig. 1a, (b, lower panel, 1^st^ lane) & (c, lower panel, 1^st^ lane)]. Neuroblasts/neural progenitors could be derived either from VZ or SVZ and typically neuroblasts of SVZs are the one that are termed as intermediate (or) basal progenitors. As the proposed study is intended to evaluate the effect of alcohol in basal progenitors, we sought to verify the E18 neuroblasts. We performed immunoblotting for Tbr2/EOMES, which is a marker for subventricular basal progenitors of neocortex. Initial screening illustrated that these E18 neuroblasts strongly express Tbr2 confirming their basal progenitor nature (Fig. 1a). Application of cAMP for 24 h stimulated neuronal differentiation which is apparent by neurite outgrowth (Fig. 1b, right) accompanied by expression of neuronal marker NSE (Fig. 1b, lower panel). The classical neural differentiation potential of these nestin-positive cells was further determined by maintaining the cells in serum free media. As expected 24 h of serum deprivation induced differentiation which was evident by neuron like morphology with spherical cell body, long processes (Fig. 1c, right) along with NF-200, a neuronal marker expression (Fig. 1c, lower panel). Based on these evidences provided, the E18 neuroblasts are indeed characterized as basal progenitor of the SVZ with potency to differentiate into neurons.

### Ethanol inhibits the proliferation of basal progenitors

**Fig. 2:**
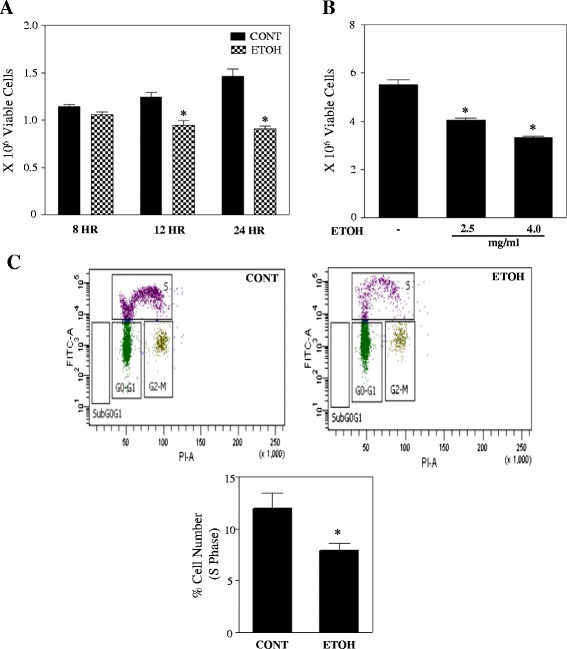
Ethanol inhibits the proliferation of basal progenitors. **a** Cells were treated with or without ETOH [4 mg/ml (86 mM)] for 8, 12 and 24 h and subsequently cells were counted by Vi-Cell cell viability analyzer. Statistical analysis was determined by one-way analysis of variance (ANOVA) and Newman-Keul’s posthoc test. *p < 0.05 when compared with untreated control. **b** A measure of proliferation index in the last cycle after 14 h treatment of ethanol in a chronic intermittent ethanol exposure (CIE) model as mentioned in materials and methods. Significance was determined by one-way analysis of variance (ANOVA) and Newman-Keul’s posthoc test. *p < 0.05 when compared with untreated control. **c** To further confirm the anti-proliferative effect of ETOH, classical BrdU based cell cycle progression analysis was performed. Cells were pulse-labeled with BrdU during the last 4 h of treatment before harvesting at 24 h. (Top): Representative scatter plots with the log FITC anti-BrdU staining versus PI staining. (Bottom): The graph illustrates the percentage of cells in synthesis phase. Sub G0-G1 phase depicts apoptotic cells. *p < 0.05 when compared with control as assessed by Student’s t-test

Ethanol is known to inhibit the proliferation of adult neural progenitor cells and suppress neurogenesis [34, 35]. However, these studies have focused on neural stem cells and apical progenitors. Notably, the effect of ethanol induced cellular alterations in basal neuroprogenitor cells (BPs) that detach from ventricular surface and proliferate to augment the growth and control of cortical size is still poorly understood. Thus, we first determined the effect of ETOH of BPs growth using trypan blue dye exclusion using Vi-CELL analyzer. In the acute ETOH exposure model, the number of viable neuroblasts were significantly decreased (*p* < 0.05) after 12 and 24 h of ETOH treatment without any change in the percentage of trypan blue-stained dead cell (Fig. 2a). Similarly, chronic intermittent ETOH paradigm (CIE) produced a dose dependent significant decrease in cell number (*p* < 0.05) (Fig. 2b). Anti-proliferative effect of ethanol was further assessed by BrdU incorporation using flow cytometry (Additional file 1: Figure S1). BrdU is a synthetic analogue of nucleotide thymidine, which incorporates into the DNA of proliferating cells during the synthesis phase of the cell cycle. Cells that have taken BrdU instead of thymidine are detected by BrdU antibody thus serving as a marker for proliferation. As shown in Fig. 2c, ETOH significantly reduced the number of cells in synthetic (S) phase as indicated by the decrease in BrdU incorporation compared to the untreated control (*p* < 0.05). Collectively, these results suggest that ETOH suppresses the growth of basal neuronal progenitors with no apparent cytotoxicity to BPs.

### Ethanol does not induce apoptosis or autophagy in basal progenitors

**Fig. 3:**
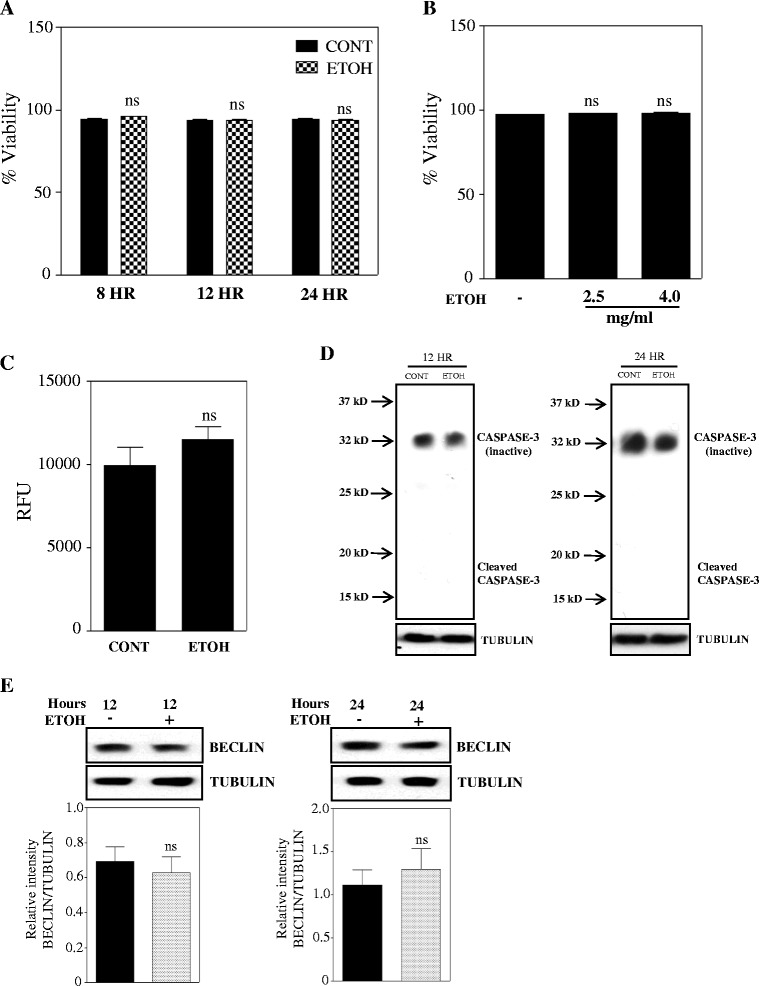
Ethanol does not induce apoptosis or autophagy in basal progenitors. **a** Cells were exposed with 4 mg/ml ETOH for 8, 12 and 24 h and subsequently percentage viability was determined using Vi-Cell analyzer. **b** Percentage viability for chronic intermittent ethanol exposure (CIE) model. Data were analyzed by one-way analysis of variance (ANOVA) and Newman-Keul’s posthoc test and no significant difference was noted among the groups. **c** Cytotoxic effect of ethanol was assessed using ApoTox-Glo Triplex assay kit and the graphs represent relative fluorescence unit from dead cells. **d** Equal amount of protein lysates from either control or 12 and 24 h ETOH treated samples were subjected to immunoblot analysis for detection of activation of caspase-3 (cleaved caspase). Tubulin was used as a loading control. **e** Western blot analysis was performed on cell lysates treated with or without ETOH for 12 and 24 h to determine the protein levels of autophagosome initiation marker, beclin 1. Bottom panel demonstrates relative intensity of beclin 1 over loading control tubulin. A representative immunoblot image is provided for panels **d** and **e**. For panel **c** and **e**, Student’s t-test was performed and ns indicate not significant when compared with control

Next in order to verify if the reduced number of cells is a resultant of induction of cell death, we determined the percentage viability with and without ETOH treatment by Vi-CELL analyzer. Fig. 3a and 3b illustrates that the percentage viability of ETOH treated cells is same as controls (~98 %) indicating that the cell viability was not altered by acute or chronic ETOH exposure despite reduced survival fraction (Fig. 2a). We next evaluated if ETOH is cytotoxic to basal progenitors by performing CytoTox-Fluor assay that measures the protease activity from dead cells using cell impermeable, fluorogenic, bis-alanylalanyl-phenylalanyl-rhodamine 110 (bis-AAF-R110) substrate that does not generate any signal in intact live cell. No significant cytotoxicity was observed in basal progenitors treated with ETOH (4 mg/ml) when compared to untreated control (Fig. 3c).

Further we evaluated if there are any signs of induction of type I programmed cell death (PCD) by assessing caspase-3 cleavage, an essential step in the activation of executioner cysteine protease using immunoblotting. No apparent change in the cleaved caspase-3 fragments was noted after 12 or 24 h of ETOH treatment (Fig. 3d). These results imply that type I PCD (apoptotic) signaling was unaltered by ETOH in basal neuronal progenitors. Under nutrition deprivation and certain cellular stress, the cells that have evaded from type I PCD have been shown to undergo type II PCD known as autophagic cell death. Hence, we determined if ethanol initiates any change in autophagic based mechanisms by analyzing the protein levels of beclin 1, a critical regulator of autophagosome formation and autophagy signaling pathways. Western blot analysis revealed no significant changes in protein levels of Beclin 1 in response to ETOH treatment (Fig. 3e). Additionally, ETOH treatment showed absence of cells in the sub G0-G1 cell region (Fig. 2b; dot plot), a general indicator of cells undergoing apoptosis. Thus the expression of key apoptosis and autophagy related proteins along with viability and cytotoxicity measures indicate that the ethanol-induced antiproliferative effect of basal cortical neural progenitors does not appear to involve apoptosis or autophagy.

### Ethanol arrests cell cycle progression by decreasing cyclin D1 and Rb phosphorylation in basal progenitors

**Fig. 4:**
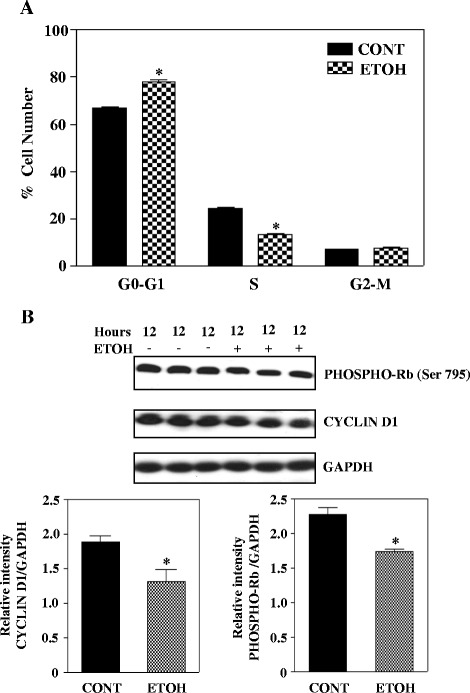
Ethanol decreases cyclin D1 and phospho-Rb protein expression and alters cell cycle progression in basal progenitors. **a** Cell cycle analysis was performed on untreated and ethanol treated cell samples with propidium iodide staining. Results were analyzed by flow cytometry to determine the percentage of cells in each phase of the cell cycle. Statistical significance was obtained by two-way analysis of variance (ANOVA) with Bonferroni post hoc tests. *p < 0.05 when compared with untreated control. **b** Cultured cells were treated with or without 4 mg/ml ETOH for 12 h. Following treatment the protein levels of phospho-Rb and cyclin D1 were determined by Western blot analysis. Lower panel depicts the quantification of protein signals using densitometric scanning. Statistical analysis was performed by Student’s t-test and *p < 0.05 when compared with control

The process of proliferation is closely connected to cell cycle regulation and since ETOH is known to interfere with the cell cycle machinery, we asked if the cytostatic effect of ETOH is a resultant of altered cell cycle kinetics. Quantitative cell cycle FACS analysis revealed that ETOH significantly increased the proportion of cells in G0-G1 phase with a concomitant decrease in S phase cells compared to control (*p* < 0.05, Fig. 4a). This implies that ETOH evokes a G0-G1 arrest of BPs. Expression and accumulation of cyclin D1 and phosphorylation of Rb are critical determinants of G1/S phase transition. During early G1, the high levels of cyclin D1 and its interaction with cyclin dependent kinase 4/6 phosphorylates the retinoblastoma (Rb) gene dissociating E2F1/DP heterodimers. This leads to activation of transcription factor, E2F and its target genes that are involved in triggering the transition of cells from G1 to S phase allowing progression of cell cycle. Hence to gain insight into the mechanism of G1 phase arrest by ETOH, we analyzed the protein levels of cyclin D1 and phosphorylation state of Rb in basal progenitors. As shown in Fig. 4b, ETOH significantly (*p* < 0.05) reduced cyclin D1 protein expression with an associated decrease in phosphorylation of Rb (*p* < 0.05). These results indicate that ETOH alters cell cycle dynamics in conjunction with expression of cell cycle regulators to impair proliferation of BPs.

### In utero ethanol exposure significantly decreases Tbr2 and cyclin D1 positive cell number in cerebral cortex of embryos

**Fig. 5:**
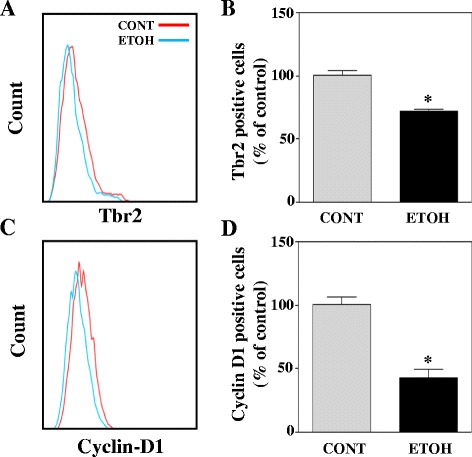
In utero ethanol exposure decreases Tbr2 and cyclin D1 positive cell numbers in fetal cerebral cortex. Pregnant Sprague-Dawley rats were administered ETOH 3.5 g/kg body weight or iso-caloric dextrose (CONT) by gastric intubation by gavage at 12 h interval for 3 days starting embryonic day 15 (E15) and sacrificed at embryonic day 18 (E18). Embryos were removed and the cerebral cortex was dissected from the rest of the brain. Tissues were isolated into single cells by mechanical disruption and were dual-labeled with Tbr2 antibody conjugated with PE and cyclin D1 antibody conjugated with FITC. **a** Tbr2-positive cells were identified by gating on the single parameter PE histogram. **b** Tbr2-positive cells were further analyzed for the expression of cyclin D1 by gating on single parameter FITC histogram. (**a & c**, analyzed by FlowJo software). **c** The bars represent percent Tbr2-positive cells in controls and ethanol treatment (**d**) Percent positive cyclin D1 cells within Tbr2-positive cells. (**b & d** analyzed by FACSDiva software). Data was analyzed by Student’s t-test. *p < 0.05 when compared with control. *n* = 4

Having shown that ethanol inhibits the proliferation of basal progenitors in vitro, we next examined whether the ethanol-induced decrease in proliferation is also replicated in vivo. In utero ethanol exposure led to a reduction in the number of cells expressing the basal progenitor marker, Tbr2 which is indicated by the backward shift (blue peak) in median fluorescence of PE-positive (Tbr2) (Fig. 5a). Further quantification revealed that maternal administration of ETOH significantly reduced (*p < 0.05) the Tbr2-positive cells by ~ 30 % (Fig. 5b) in fetal cerebral cortices. Additionally, the median fluorescence of FITC-positive cells (denoting cyclin D1 expression) in Tbr2 gated population was significantly diminished by ~50 % in response to ETOH as compared to iso-caloric control (Fig. 5c and 5d). Therefore, consistent with in vitro observation, ETOH exposure indeed impairs the proliferation of basal progenitors in vivo likely through alterations in cyclin D1-associated cell cycle events.

## Discussion

Consumption of alcohol during the sensitive periods of neurogenesis leads to developmental brain disabilities associated with FAS [36]. Several studies have shown that alcohol affect the critical processes of neurogenesis during the early phases of brain development [37]. However majority of the studies focused on neural stem cells, radial glia cells (RGCs) and apical progenitors. In the current study, we used spontaneously established Tbr2-positive, neuron-restricted basal neuroprogenitors (intermediate) from embryonic day 18 day of rat cerebral cortices alongside in utero binge alcohol model to investigate the mechanism underlying ethanol induced abnormal corticogenesis. Basal progenitors (BPs) are known to be the precursors for projection neurons that populate the various cortical layers that selectively express Tbr2 [20, 38, 39]. Our results demonstrate for the first time that ethanol impairs the proliferation of basal progenitor cells in vitro (acute and CIE). In addition, in utero alcohol exposure significantly decreased the number of Tbr2 expressing cells (Fig. 5a) indicative of dysregulated genesis of basal progenitors. Our results are consistent with an elegant report that demonstrated a decrease in Tbr2 immunoreactivity in the cortical VZ/SVZ regions of fetal human and mouse slice cultures exposed to ETOH [40]. Further, another study by Mo et al. [41] illustrated that ETOH affects the expression of Pax6 in RGCs resulting in reduced generation of Tbr2+ cells and young neurons. The difference could be ascribed to the fact that the cells used in the Mo et al., [41] study is 95 % radial glia origin as opposed to the basal progenitors used in the current study (strong Tbr2 expression; Fig. 1). Loss of functional mutation in human Tbr2 gene has been demonstrated to cause microcephaly and polymicrogyria (abnormal gyrus formation) [42] and the former is one of the abnormal phenotypes observed in FASD. Thus the resulting ETOH-induced cytostasis of BPs without any apparent cytotoxicity could affect the mainstream corticogenesis as seen in FAS model and Tbr2 deletion transgenic study [43].

Earlier, we and others have shown that ETOH induces cell death by activating caspase-3 in rat fetal cortical neurons and cerebral cortex [29, 44–46]. On the other hand, studies have shown that rat and human neural progenitor cells are resistant to ETOH-induced apoptotic cell death [47–50]. In the present study, we did not observe cytotoxicity or caspase-3 activation or any morphological signs reflective of apoptosis that are in agreement with prior findings. Further, with no change in beclin 1 levels, the involvement of autophagic based mechanisms on the growth inhibition of BPs induced by ETOH is also precluded. Overall, these results suggest that cortical BPs are resistant to acute treatment of ETOH-induced cell death and further indicating that the effect of ETOH on basal progenitor is indeed growth inhibitory. Compelling evidence of the significance of cell cycle derangements as a mechanism in ETOH-induced growth suppression of BPs is presented. We established that 86 mM ETOH inhibits cell proliferation by interfering with the cell cycle at G1-S transition. Of more relevance, a blood alcohol concentration (BAC) of ~180 mg/dl has been shown to increase the duration of the cell cycle by 29 % in the telencephalic ventricular zone of rats [51]. An in vitro study by Mikami et al. [52] showed that 10 or 50 mM ETOH induced both transient and permanent cell cycle arrest at G2-M and G1-S phase respectively in mouse fibroblastic cells. However, our cell cycle FACS experiments revealed that prior to entering G2 and subsequently to M phase, the BPs were blocked at G1-S transition. The difference could be due to diversity in the cell types and level of ROS generated as a result of dose used. Typically, sub-lethal doses of H_2_O_2_ and hyperoxia induced oxidative stress has been shown to induce permanent cell cycle arrest [53, 54]. Analogous to our current finding, earlier study by Li et al. [55] has reported that 400 mg/ml ethanol exposure lengthens the duration of both the total cell cycle and S-phase in cerebellar granular progenitors. Of importance, ROS events associated with hyperoxia, X-rays or UV irradiation induces S phase arrest to a greater degree [56–58] and since ETOH can converge at the point of ROS generation with these stressors, a suppression of proliferation by obstruction of the G1-S cell cycle events is highly plausible with ETOH in BPs. However, multi-phase arrest including G2-M could not be completely disregarded as perturbations of cell cycle events at S phase is shown to cause cell cycle arrest at G2/M [59].

Cell cycle progression involves sequential and co-ordinated regulation of cyclin-dependent kinases (CDKs) along with large family of cyclins that interact with and activate CDKs. Cyclin Ds are the key regulators that drive G1-S phase transition by interacting with CDK2 and phosphorylating Rb [60]. Rb is a repressor of E2F and hyerphosphorylation of Rb relieves E2F suppression which then transcriptionally promotes several of its targets and promotes phase transition of G1-S [61]. We demonstrate that ETOH decreased the expression of cyclin D1 with a corresponding reduction in phosphorylation of Rb in vitro (Fig. 4b). In addition, in utero ETOH administration decreased the percentage of cells staining positive for cyclin D1 in Tbr2-gated population representing basal neuronal precursors (Fig. 5b). Consistent with our findings, previous study has shown that ETOH induced impairment in cyclin D1 expression was associated with cell cycle arrest at G1 phase [62]. This suggests that ETOH induced dysregulation of cell cycle progression and growth is associated with cyclin D1 expression decrement in Tbr2-positive basal progenitors.

## Conclusion

**Fig. 6:**
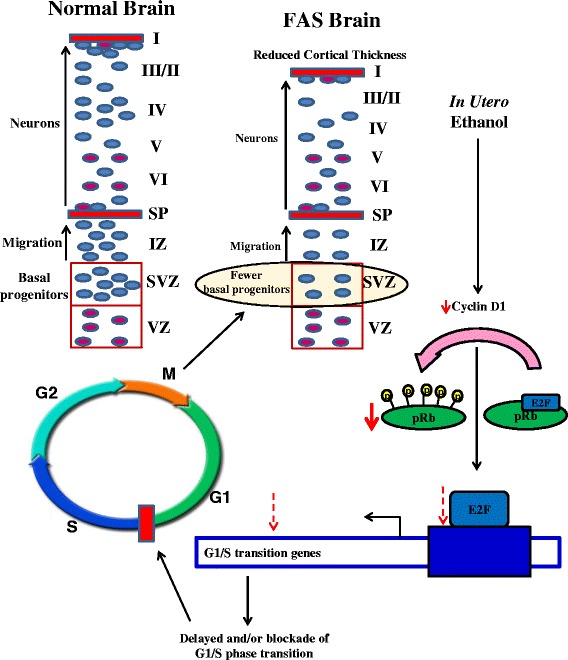
Proposed mechanism of alcohol-induced damage to developing cerebral cortex.  Indicates decreased protein levels.  not tested but proposed alteration in E2F and target genes.  Delayed progression and/or blockade of cell cycle from G1  S. SVZ- subventricular zone, VZ- ventricular zone, IZ- intermediate zone, SP- superficial portion of the intermediate zone, cortical layers I, II, III, IV, V and VI

Maternal alcohol consumption during pregnancy leads to fetal alcohol spectrum disorder characterized by CNS abnormalities. Though prior studies have addressed the growth inhibitory effect of ethanol on ventricular apical progenitors, there is a lacuna in understanding the effect of ethanol on basal progenitor, which primarily generates upper cortical layers. In the current study, we have demonstrated that both in vitro and in utero ethanol exposure impairs the basal progenitor proliferation, an important event that governs the competency of progenitor production and its fate. Further, we showed that ethanol induced obstruction of cell cycle progression at G1-S phase marked by decrease in cyclin D1 and phospho-Rb expression as a plausible mechanism for the cytostatic effect of ethanol (Fig. 6). Further investigation is warranted to illuminate the involvement of cyclin D1 and cyclin E, essential for the G1-S phase transition in ethanol-induced inhibition of proliferation, which will ultimately enhance our understanding of mechanisms that damage the developing brain.

## Additional file

Additional file 1: Figure S1. Ethanol dose dependently decreases cell proliferation: BrdU incorporation as an index of cell proliferation was determined after exposing the cells with 2.5 mg/ml (56 mM) and 4 mg/ml (86 mM) ETOH for 24 h. Absorbance was determined at 450 nm. Results were analyzed by one-way analysis of variance (ANOVA) and Newman-Keul’s posthoc test. **p* < 0.05 when compared with untreated control. (PPTX 43 kb)

## Abbreviations

FASD: fetal alcohol spectrum disorder
FAS: fetal alcohol syndrome
ETOH: ethanol
CONT: control
CNS: central nervous system
GAPDH: glyceraldehyde-3-phosphate dehydrogenase
GFAP: glia fibrillary acidic protein
NF: neurofilament
BrdU: 5-bromo-2’-deoxyuridine
cAMP: cyclic adenosine monophosphate
CIE: chronic intermittent ethanol exposure
Tbr2: T-box brain protein 2
EOMES: Eomesodermin
PBS: phosphate buffered saline
CDK: cyclin dependent kinase
ANOVA: analysis of variance
