# Supplementary figures and images for: Ethanol induces cytostasis of cortical basal progenitors
Source: J Biomed Sci. 2016 Jan 19;23:6. doi: 10.1186/s12929-016-0225-8 (PMC4717586; doi:10.1186/s12929-016-0225-8)

## Slide 1
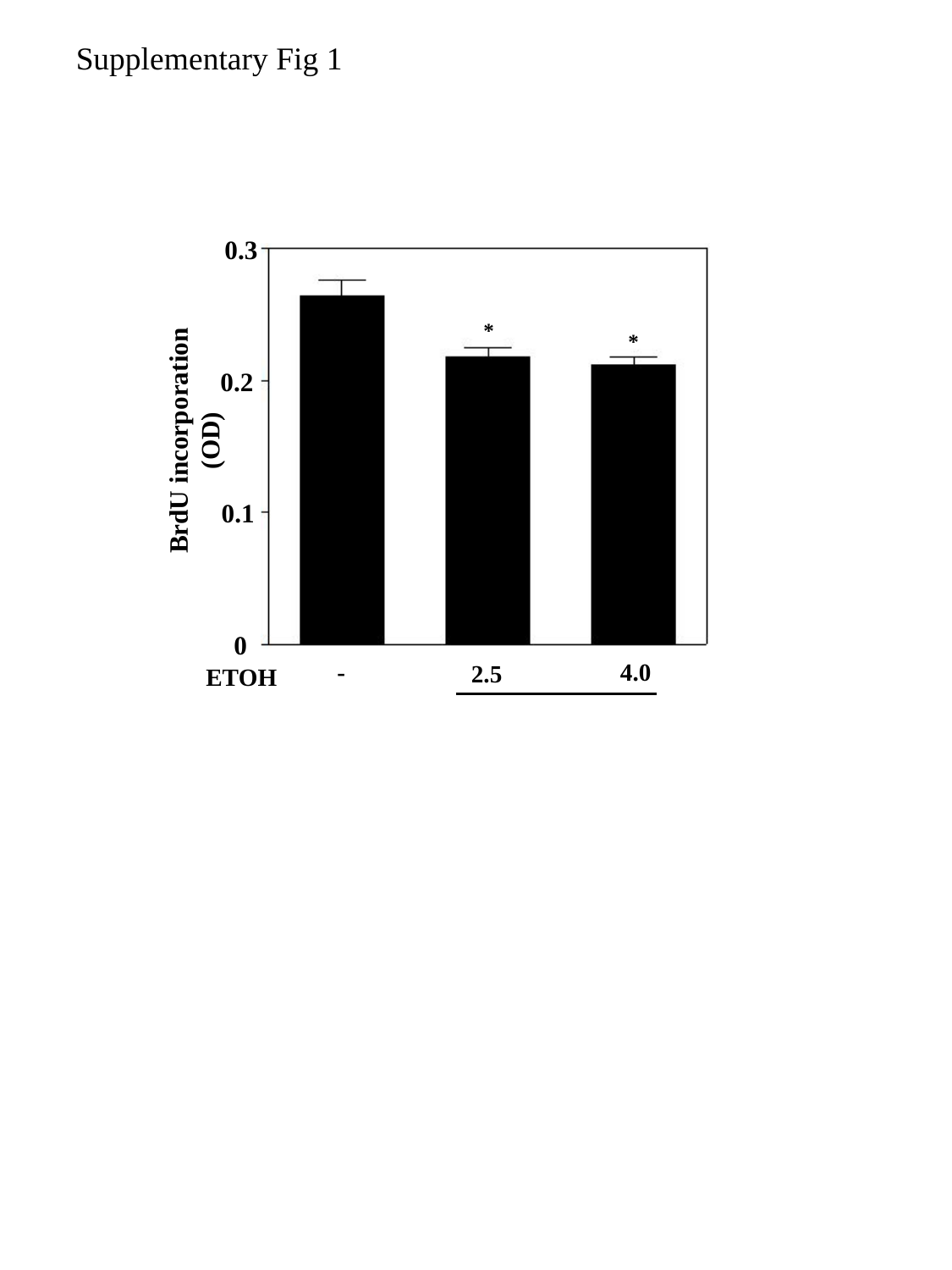

Supplementary Fig 1
0.3
*
*
0.2
BrdU incorporation (OD)
0.1
0
4.0
-
2.5
ETOH

Supplement: Additional file 1: Figure S1. — Ethanol dose dependently decreases cell proliferation: BrdU incorporation as an index of cell proliferation was determined after exposing the cells with 2.5 mg/ml (56 mM) and 4 mg/ml (86 mM) ETOH for 24 h. Absorbance was determined at 450 nm. Results were analyzed by one-way analysis of variance (ANOVA) and Newman-Keul’s posthoc test. *p < 0.05 when compared with untreated control. (PPTX 43 kb) [file 12929_2016_225_MOESM1_ESM.pptx]
